# Highly parallel bending tests for fungal hyphae enabled by two-photon polymerization of microfluidic mold

**DOI:** 10.3389/fbioe.2024.1449167

**Published:** 2024-11-01

**Authors:** Steffen Brinkmann, Marcel Schrader, Sven Meinen, Ingo Kampen, Arno Kwade, Andreas Dietzel

**Affiliations:** ^1^ Institute of Particle Technology, Technische Universität Braunschweig, Braunschweig, Germany; ^2^ Institute of Microtechnology, Technische Universität Braunschweig, Braunschweig, Germany; ^3^ Center of Pharmaceutical Engineering, Technische Universität Braunschweig, Braunschweig, Germany

**Keywords:** cell wall, hyphae, *Aspergillus niger*, bending stiffness, fungi-on-chip, two-photon polymerization

## Abstract

Filamentous microorganisms exhibit a complex macro-morphology constituted of branched and cross-linked hyphae. Fully resolved mechanical models of such mycelial compounds rely heavily on accurate input data for mechanical properties of individual hyphae. Due to their irregular shape and high adaptability to environmental factors, the measurement of these intrinsic properties remains challenging. To overcome previous shortcomings of microfluidic bending tests, a novel system for the precise measurement of the individual bending stiffness of fungal hyphae is presented in this study. Utilizing two-photon polymerization, microfluidic molds were fabricated with a multi-material approach, enabling the creation of 3D cell traps for spore immobilization. Unlike previous works applying the methodology of microfluidic bending tests, the hyphae were deflected in the vertical center of the microfluidic channel, eliminating the adverse influence of nearby walls on measurements. This lead to a significant increase in measurement yield compared to the conventional design. The accuracy and reproducibility of bending tests was ensured through validation of the measurement flow using micro-particle image velocimetry. Our results revealed that the bending stiffness of hyphae of *Aspergillus niger* is approximately three to four times higher than that reported for *Candida albicans* hyphae. At the same time, the derived longitudinal Young’s Modulus of the hyphal cell wall yields a comparable value for both organisms. The methodology established in this study provides a powerful tool for studying the effects of cultivation conditions on the intrinsic mechanical properties of single hyphae. Applying the results to resolved numerical models of mycelial compounds promises to shed light on their response to hydrodynamic stresses in biotechnological cultivation, which influences their expressed macro-morphology and in turn, product yields.

## 1 Introduction

Filamentous microorganisms are highly relevant to the biotechnological industry, as they can produce a broad range of substances such as organic acids, pharmaceutical substances or enzymes (Meyer et al., 2016). When grown in submerged culture, their developed macro-morphology profoundly influences their productivity (Nielsen et al., 1995; Grimm et al., 2005; Wucherpfennig et al., 2011; Krull et al., 2013), where the morphology can range from loosely dispersed mycelium up to dense agglomerates known as pellets (Walisko et al., 2015; Böl et al., 2021). Which morphology is desirable to maximize product yield varies between different strains and depends on the desired product (Pazouki and Panda, 2000; Grimm et al., 2005; Wucherpfennig et al., 2010). Therefore, a good understanding of the parameters that influence the expressed growth morphology is crucial for targeted process optimization by morphology engineering. The hydrodynamic conditions inside the reactor volume are an important factor influencing macro-morphology (Serrano-Carreón et al., 2015; Waldherr et al., 2023). If the power input in a bioreactor is too high, the latter can lead to cell damage and thus reduced productivity (Toma et al., 1991; El-Enshasy et al., 2006). On the other hand, if the agitation and aeration is too low, inhomogeneous mixing can lead to insufficient oxygen supply, which is why studies have been carried out to link hydrodynamic stress to resulting morphology and thus productivity (Lin et al., 2010; Waldherr et al., 2023). In the case of filamentous microorganisms, agitation leads to relative velocity between mycelial compounds (e.g., pellets) and liquid media, resulting in shear and normal forces acting on the hyphae (Ayazi Shamlou et al., 1994). Therefore, an accurate knowledge of their intrinsic mechanical properties is key for the accurate modelling of the mechanical response of more complex hyphal networks to such stresses. Consequently, the aim of this work is to mechanically characterize the hyphae of a representative strain of the filamentous fungus *Aspergillus niger*, which is highly relevant and widely used in white biotechnology (Cairns et al., 2018).

One established approach to measure the mechanical properties of single hyphae is atomic force microscopy (AFM) based force spectroscopy, which is also often applied to more regularly shaped cells, such as yeast or micro algae (Arfsten et al., 2010; Günther, 2015). It allows very local probing of the elasticity of the cell wall and has been applied to derive hyphal cell wall stiffness (Ma et al., 2005; Zhao et al., 2005; Zheng et al., 2017). Utilizing this high spatial resolution, Ma et al., 2005 showed that the stiffness of the cell wall of *Aspergillus nidulans* hyphae shows a harsh gradient, decreasing towards the hyphal tip. However, the very local character of AFM can also be disadvantageous, as the probed elasticity can depend strongly on the indentation location, complicating the extraction of intrinsic mechanical properties (Arfsten et al., 2010). Moreover, varying the measurement parameters, especially the AFM tip geometry, can lead to considerably large variations in measured elasticity (Wu et al., 2018).

An alternative approach to avoid the implications of local probing are global measurement methods, in which the entire cell is subjected to mechanical stress to infer its mechanical properties. Stocks and Thomas (2001) used this approach in a tensile test setup to measure the tensile strength and Young’s modulus of single hyphae of the filamentous bacterium *Saccharopolyspora erythraea* (Stocks and Thomas, 2001). While this method allows determination of the tensile strength, it requires a high degree of manual interaction with the hypha for precise positioning and immobilization, resulting in low throughput and not being feasible for *in situ* application. In single cell compression tests, whole cells are compressed by a nanoindenter equipped with a flat punch (Arfsten et al., 2009; Overbeck et al., 2015; 2023). Accounting for the cell deformation, the yielded stiffness can be used to estimate the elasticity of the cell wall. However, applying this methodology to hyphae is not straight forward because of their irregular shape. Instead of mechanically stressing the entire hypha, Chevalier et al. precisely punctured the cell wall of *A. nidulans* hyphae via photoablation, resulting in the release of turgor pressure and thus a relaxation of the cell wall. Using a previously determined turgor pressure, they measured a gradient in cell wall Young’s Modulus that increased with distance from the hyphal tip (Chevalier et al., 2023).

While the aforementioned methods do enable the measurement of the hyphal cell wall’s elasticity in some way, they require a high degree of manual interaction with the specimen and thus suffer from low throughput. A non-invasive method to globally measure the hyphal cell wall’s elasticity, which also has the potential for high parallelization is a microfluidic bending test (Nezhad et al., 2013; Amir et al., 2014; Caspi, 2014; Couttenier et al., 2022). This methodology uses flow-induced forces inside a microfluidic channel to deflect filamentous cells oriented perpendicular to the flow direction. It can be implemented quasi *in situ* during an ongoing cultivation. Nezhad et al. (2013) published the first demonstration of measuring the Young’s Modulus of the primary cell wall of *Camellia japonica* pollen tubes using this method. After conducting a proof of concept, Amir et al. and Caspi utilized comparable systems to measure the bending stiffness of *Escherichia coli* and *Bacillus subtilis* cells, thus expanding the method to bacterial cells (Amir et al., 2014; Caspi, 2014). Most recently, Couttenier et al. (2022) improved the technique by implementing a more sophisticated microfluidic design to measure the hyphae of the dimorphic yeast *Candida albicans*. In all of these works, the microfluidic system served as a platform for the actual measurement and as an incubator for the immobilization and growth of the test samples, minimizing the need for manual handling. In addition, in all previous studies that utilized the microfluidic bending tests, standard photo- and soft-lithography protocols were used for fabrication. As a consequence the filaments being measured were situated on the channel floor. This can lead to various issues, such as partial adhesion to the floor surface, which can interfere with measurements, or even complete immobilization of the hypha, making measurements impossible (Couttenier et al., 2022). This work presents a system for microfluidic bending tests that prevents interference of the channel floor with the bending measurement. For this purpose, the hyphae had to be positioned in the vertical center of the channel. To enable true 3D structuring and at the same time achieve the necessary high resolution for manipulating single fungal hyphae, the microsystems were fabricated by a two-photon polymerization (2 PP) assisted molding process. Moreover, a multi-material approach for the 2 PP process was required to integrate micrometer feature resolution in a structure spanning multiple millimeters.

## 2 Materials and methods

### 2.1 Microfluidic chip design

**FIGURE 1:**
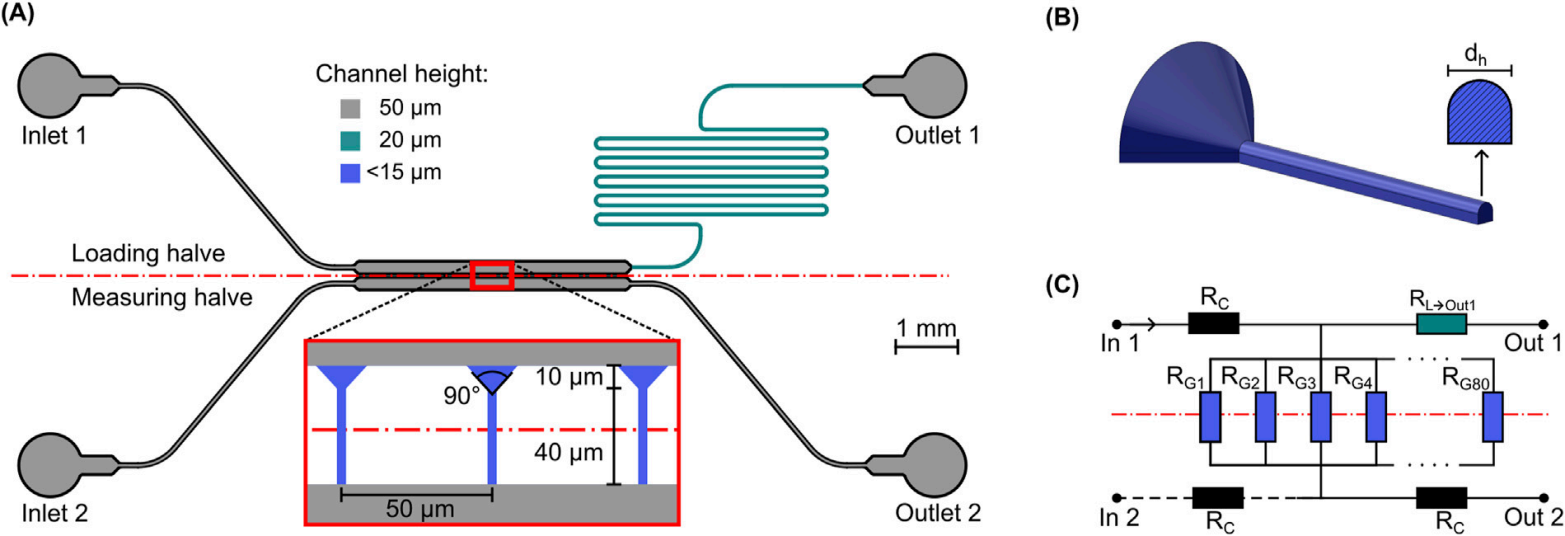
Schematic overview of the system design. **(A)** Top view of the microfluidic system layout with a zoomed view of the middle section with the growth channels. The separation of the loading and measurement halves is indicated by the dashed red line, different channel colors show different channel heights. **(B)** Perspective view of the 3D-shape of a growth channel and cross-section of narrow part. **(C)** Simplified fluidic equivalent circuit diagram used for the channel dimensioning and estimation of average chamber pressure.

The basis for the presented system design is an adaption of the design for *C. albicans* hyphae described by Couttenier et al. (2022). Briefly, to accommodate for the two main tasks of spore immobilization and measurement, the system was designed in two halves - the loading and the measuring halve - each containing a main chamber, either the loading chamber or the measurement chamber (see Figure 1A). Inside the former, spores were immobilized and subsequently incubated to germinate and grow hyphae. Inside the latter, the probing flow subsequently deflected the hyphae growing into it. Both system halves had separate inlets for infusion with either spore suspension or liquid medium, as well as dedicated outlets. To immobilize and guide growth, 80 equidistantly spaced growth channels with a cross-section matching that of a hypha ( $d_{h}\approx 2.8$ µm) connected the two main chambers, as shown in the inset in Figure 1A. These channels acted as both a cell trap for the spores and a spatial guide for growth into the measurement flow. To ensure good shape conformity with the spherical spores, we designed the entrance of the growth channels to be funnel-shaped with an opening angle of 90°. The funnel shape was fabricated as a 3D shape using 2 PP fabrication. Because of the high shape conformity with the spherical spores this eliminated the need for additional measures to prevent slip of the immobilized spore/hypha during measurements, as described in previous publications (Nezhad et al., 2013; Couttenier et al., 2022). To prevent undercuts during the replication molding process, the substrate facing side of the 3D shape was projected onto the substrate, as illustrated in Figure 1B. During spore loading, it was crucial to ensure that a significant portion of the total spore-laden volume flow passed through the traps. This was achieved through an additional fluidic resistance between the loading chamber and the loading outlet in the form of a serpentine channel with a reduced cross-section. The length of the flow path was designed to achieve an overall fluidic resistance that is 20 times the value of the parallel fluidic resistances of all growth channels at the start of the loading step, assuming no channels are blocked by spores. Figure 1C shows the equivalent fluidic circuit used to calculate the required resistance. As a result, the ratio of the volume flow through the growth channels towards Outlet 2 and through the serpentines towards Outlet 1 was predicted to be approximately 20 at the beginning of the loading process. With each growth channel blocked by a spore, this volume flow ratio decreases, while simultaneously the average absolute pressure in the loading channel increases. However, only up to a maximum when all channels are blocked. This method allowed safe loading without risking damage to the microfluidic device from overpressure caused by spores completely clogging the flow path.

### 2.2 2PP of the microfludic mold

**FIGURE 2:**
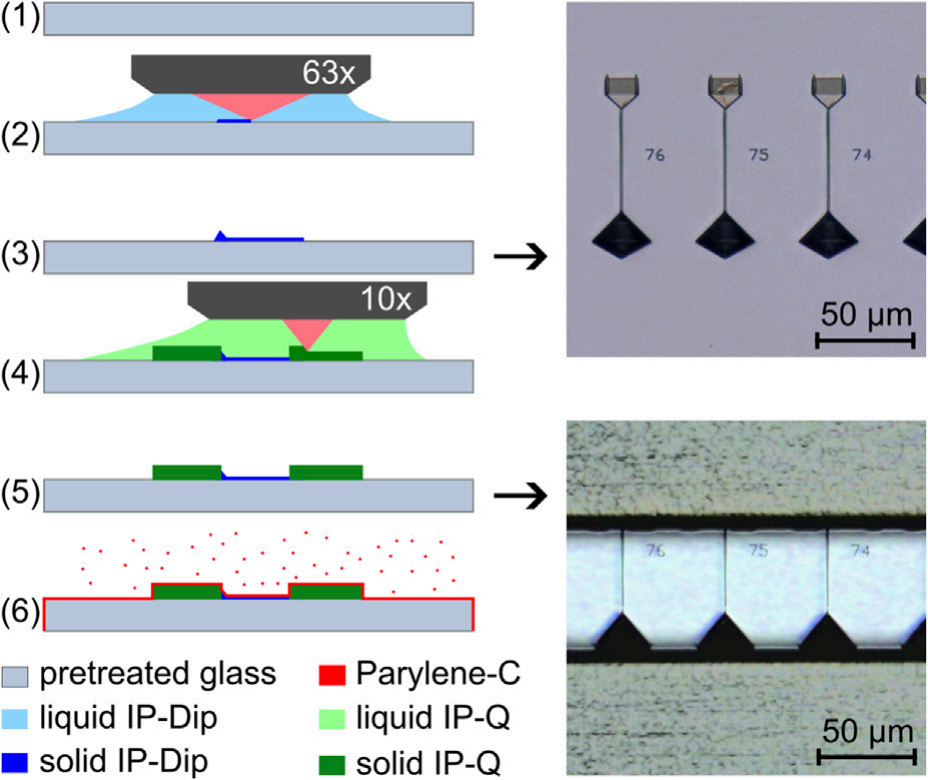
Fabrication steps of the negative mold by two-photon polymerization viewed from the side with white light microscopy images of the top view of the growth channel structures after the 63x-process and with the added channel structures after the 10x-process.

Figure 2 illustrates the process of creating the negative mold for soft-lithography replica molding through 2 PP. A previously developed process for the production of dry adhesive structures (Busche et al., 2020) was adapted for this purpose. The glass substrate (*Borofloat 33, Schott, Mainz, Germany*) was first rinsed with acetone, ethanol, and millipore water. After blow-drying with nitrogen, the substrate was coated with an approximately 30 nm thick film of indium tin oxide (ITO) via sputtering (*Laborsystem LS 440 S, Von Ardenne Anlagentechnik GmbH, Dresden, Germany*) to facilitate automatic interface finding during the 2 PP process. Next, the ITO-coated substrate was treated with oxygen plasma (*Zepto, Diener, Ebhausen, Germany*) for 60 s at 200 W and 100 Pa, and then silanized with 3-trimethoxysilylpropyl-methacrylate (TMSPM, *Sigma-Aldrich*, *St. Louis*, *United States*) to promote structure adhesion as described by (Garoff and Ansorge, 1981). This step prevented delamination of the 2 PP structures during development and drying. After silanization (1), the negative structure of the channel network was printed using a 2 PP workstation (*Photonic Professional GT1, Nanoscribe GmbH, Eggenstein-Leipoldshafen, Germany*) equipped with a Ti:Saphire laser emitting 100 fs pulses at a central wavelength of 780 nm and a fixed rate of 80 MHz. To make the best out of the process’s full resolution while keeping printing times reasonable, the fabrication was divided into two sub-processes. In the first process, we used a 63x-objective (*NA 1.4, Carl Zeiss AG, Oberkochen, Germany*) to polymerize the finest structures (2). We used the commercially available photoresist IP-Dip (*Nanoscribe GmbH, Eggenstein-Leipoldshafen, Germany*) with a minimal radial feature size of less than 1 µm. To maintain a consistent structure height in accordance with the design, we utilized automatic interface detection for each individual structure. Following this 63x process, the structures were developed by carefully immersing the substrate in propylene glycol monomethyl ether acetate (PGMEA, *Sigma-Aldrich*, *St. Louis*, *United States*) for 15 min, followed by a rinse in isopropanol for 120 s and a gentle blow-dry with nitrogen. This yielded the fine structures of the growth channels (3). In the second 2 PP-subprocess, we used a 10x-objective (NA 0.3, *Carl Zeiss AG, Oberkochen, Germany*) with the photoresist IP-Q (*Nanoscribe GmbH, Eggenstein-Leipoldshafen, Germany*) to print the larger features of the system on top of the fine structures using (4). The result was the complete negative structure of the channel system (5). To allow good detachment from the mold, we used a parylene coater (*nttf coatings GmbH, Rheinbreitbach, Germany*) to deposit a 1 µm thick layer of Parylene-C on top of the structures (6). This uniformly deposited coating introduced a constant reduction of structure aspect ratio (height to width), which was accounted for in the initial design.

### 2.3 Microfluidic chip fabrication

**FIGURE 3:**
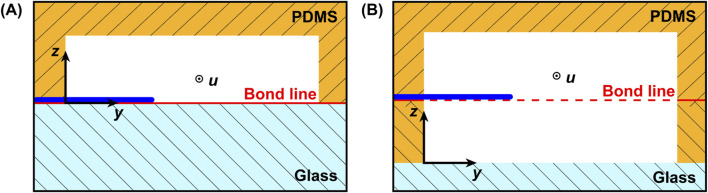
Schematic cross sectional view of the two system configurations with the hypha either **(A)** on the floor (floor configuration) or **(B)** in the vertical center (center configuration) of the measurement chamber. The bond line for both systems is depicted in red.

In order to obtain the positive microfluidic channels, the printed structures were molded with poly-dimethylsiloxane (PDMS). Liquid PDMS precursor and the corresponding crosslinker from the PDMS production set (*Sylgard 184, Dow Chemical, Midland, United States*) were mixed at a 10:1 weight ratio, degassed, and poured over the negative mold. After curing for 1 h at 70°C on a hot plate, the structured PDMS was peeled off the mold. The final assembly of the PDMS slab was conducted according to the desired system configuration. Two configurations were fabricated: the *floor configuration*, which represented a design corresponding to the current state of the art, and a second novel *center configuration*, allowing hyphae without floor contact to be exposed to the probing flow. A cross-section view of both systems is shown in Figure 3. In the floor configuration, the hyphae resided on the floor of the measurement chamber (Figure 3A), while in the center configuration it could be located in the vertical channel center, achieving a symmetrical flow around it (Figure 3B). For the floor configuration, the PDMS slab was punctured with a 1 mm autopsy punch for the in- and outlets before being permanently bonded to a glass chip through oxygen plasma treatment in a low-pressure plasma chamber (*Zepto, Diener, Ebhausen, Germany*), sealing the channels. The bonding was finalized by 4 hours at 40°C on a hot plate. Alternatively, to fabricate systems in center configuration, the PDMS slab was aligned with and bonded to a second system halve, containing the mirrored cavity of the measurement chamber. This second system halve was fabricated by spin-coating a layer of PDMS onto a glass wafer. The PDMS layer thickness was adjusted precisely to match the desired channel height, positioning the hypha in the vertical channel center. Using a femtosecond laser work station (*microSTRUCT c, 3D Micromac AG, Chemnitz, Germany*) equipped with a YB:KGW solid-state laser (*Pharos, Light Conversion, Vilnius, Lithuania*) we then selectively ablated the PDMS layer. In a subsequent laser process, through holes with a diameter of 1.2 mm were drilled through both the PDMS and the glass, resulting in the inlets and outlets. The parameters for both laser processes are listed in Table 1. After another plasma treatment (*Zepto, Diener, Ebhausen, Germany*) of both PDMS surfaces, alignment of both system halves was carried out in a mask aligner (*EVG 420, EV Group, St. Florian am Inn, Austria*). Permanent bonding was achieved through oxygen plasma treatment, as described above. As the same molded PDMS slab was used, the channel system and the cell traps where identical for both configurations, except for the measurement chamber height which was doubled.

**TABLE 1:** Parameters for femtosecond laser ablation processes.

Process	Channel (PDMS selective)	Through holes (PDMS + Glass)
Wavelength	515 nm	1,030 nm
Pulse rate	100 kHz	600 kHz
Average power	0.7 W	9 W
Scan speed	100 mm/s	2000 mm/s
Number of scans	2	2

### 2.4 Fluid handling

**FIGURE 4:**
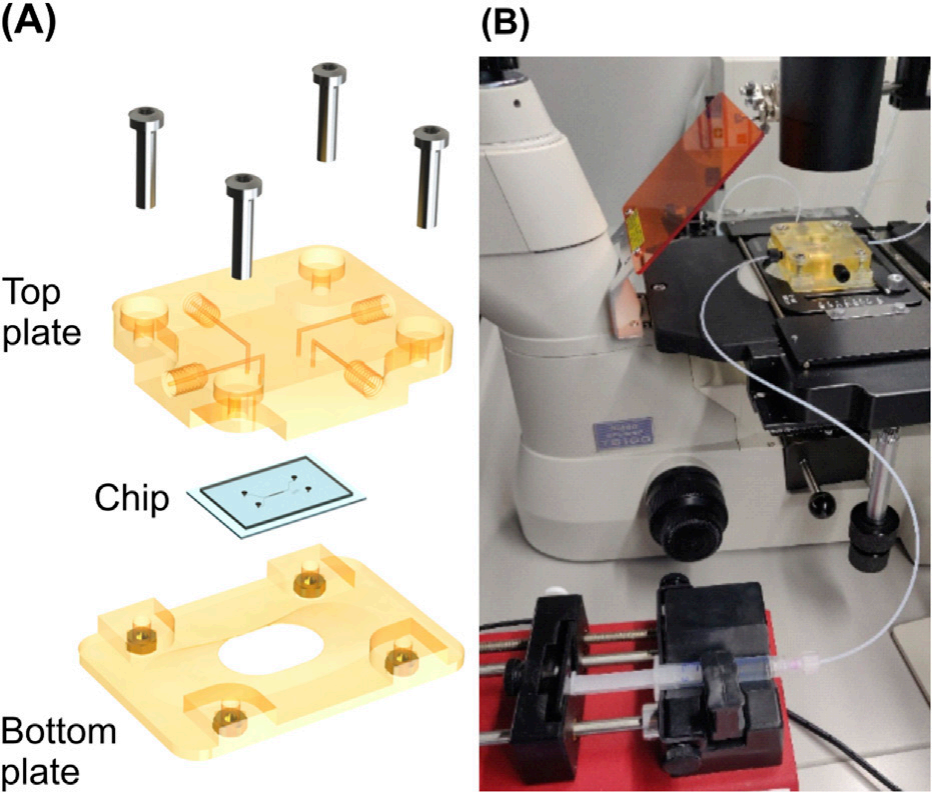
Measurement set up. **(A)** Chip holder used for fixating and interfacing of the chip during fluidic experiments. **(B)** Set up for bending experiments with a connected syringe pump in the foreground and the chip holder installed on the stage of the inverted microscope in the background.

To be able to apply fluid flow to the microfluidic chip, we designed a chip holder using Solidworks CAD software (*Dassault Systems, Vélizy-Villacoublay, France*) and 3D-printed it with a high-precision inkjet printer (*Agilista, Keyence, Osaka, Japan*). The chip holder comprised two plates between which the microfluidic chip was clamped, as shown in Figure 4A. Fluidic connection channels with a diameter of 1 mm were integrated into the top plate, which contacts the PDMS side of the system. These channels were arranged to interface the system in- and outlets. Female M6 fluidic connector ports were located at the other end of these channels, enabling the connection of PTFE tubing to the microfluidic chip. These were connected to syringes installed in syringe pumps (*AL-1000, World Precision Instruments, Sarasota, United States*). The syringe pumps and the assembled chip holder are shown in Figure 4B. For the loading flow, 3 mL (*Norm-Ject, Henke-Sass Wolf GmbH, Tuttlingen, Germany*) syringes were used, while for the measurement flow we chose a 2.5 mL glass syringe (*SETonic GmbH, Illmenau, Germany*).

### 2.5 CFD simulation

In order to enhance comprehension of the fluid-induced forces, we conducted a CFD simulation of the measurement flow around the hypha using the software ANSYS Fluent (*V. 2023 R2*, *Ansys Inc., Cacnonsburg, Pennsylvania, United States*). We used a model of a 500 µm long section of the measurement chamber with a rectangular profile and a rigid hypha positioned in the vertical channel center. We further selected velocity inlet and outflow as the inlet and outlet boundary conditions, respectively. The walls were modelled without slip and a symmetry condition was applied at the symmetry plane of the geometry to reduce computational cost. The geometry was discretized using a tetrahedral mesh, with refinement of mesh size on and near the surface of the hypha. A fully laminar flow model was used for the calculations.

### 2.6 Flow measurements

For the purpose of quantifying the forces exerted on the hyphae by the flow and to verify the theoretical flow profile, we investigated the measurement flow using Micro Particle Image Velocimetry (µPIV). We measured the particle-laden flow in the xy-plane over the entire height of the measurement chamber in z-steps of 3 μm to assess the entire flow field. To ensure good agreement of particle flow paths and flow stream lines, fluorescent particles with a mean diameter of 0.86 µm (*Fluoro-Max, Thermo Fisher Scientific Inc., Waltham, United States*) were chosen. The μPIV system used in this study (*FlowMaster, LaVision, Göttingen, Germany*) was equipped with a Nd-YAG double-pulse laser emitting at a wavelength of 532 nm at a pulse duration of 5 μs. The maximum double pulse repetition rate is 25 Hz. For each z-position, 20 individual measurements were taken, each consisting of two double frames. The time interval between the double frames was adjusted within the range of 10–100 μs, depending on the applied volume flow. This resulted in an offset of approximately 10 pixels between the double frames. To extract the velocity field across a channel cross-section, the PIV evaluation software DaVis 10 (*LaVision, Göttingen, Germany*) was used to evaluate the 20 single measurements per z-position by applying the implemented sum of correlation algorithm (Meinhart et al., 2000). The results for the individual z-positions were then stacked to obtain the flow field.

### 2.7 Fungal strain and growth media

The *A. niger* strain SKAn1015 (Zuccaro et al., 2008) was used as the organism in this study due to its extensive history of research on various aspects of its cultivation and biology (Priegnitz et al., 2012). To produce the spore suspension, spores were collected from a sporulating agar plate using a cotton swab and then spread onto a fresh agar plate. The newly inoculated agar was then incubated at 37°C for 4 days until excessive sporulation was visible. To obtain a spore suspension, the plate was flooded with a 0.9% NaCl solution and gently scrubbed with another cotton swab to loosen the spores. Next, a filtration step was performed using Miracloth filtration paper (*Merck*, pore size 22–25 µm) to remove any mycelial compounds or other unwanted contaminants from the agar plate. The spore suspension obtained was stored at 4°C for a maximum of 7 days before being used in experiments. For cultivation, a minimal medium containing 22 g L^−1^ glucose as carbon source, 0.5 g L^−1^ MgSO_4_·7H_2_O, 20 mL L^−1^ salt solution (26.1 g L^−1^ KCl, 74.8 g L^−1^ KH_2_PO_4_ and 250 g L^−1^ NaNO_3_) and 1 mL L^−1^ trace element solution (10 g L^−1^ EDTA, 4.4 g L^−1^ ZnSO_4_ 7H_2_O, 1 g L^−1^ MnCl_2_ 4H_2_O, 0.315 g L^−1^ CuSO_4_ 5H_2_O, 2.5 g L^−1^ FeSO_4_·7H_2_O, 0.32 g L^−1^ CoCl_2_ 6H_2_O, 1.47 g L^−1^ CaCl_2_ 2H_2_O CaCl_2_·2H_2_O and 0.22 g L^−1^ (NH_4_)6Mo_7_O_24_ 4H_2_O) was used. The carbon source and the other ingredients were sterilyzed separately in an autoclave before mixing. Afterwards, the whole solution was adjusted to a pH of 3 with HCl (3 M).

### 2.8 Procedure for conducting and evaluating bending tests

Spores were loaded into a microfluidic chip installed in the chip holder. For this a spore suspension of 10^6^ spores per mL in minimal medium was used to infuse spores with a syringe pump at an average flow velocity of 3 mm s^−1^. After the first spores had entered the loading chamber, the syringes were exchanged and the infusion through the loading inlet was continued with filtered minimal medium. The immobilized spores where incubated at 37°C. A constant low infusion rate of 5 μL h^−1^ on the loading and measuring side ensured that no negative pressure gradient from measuring chamber to loading chamber would result in the dislodging of immobilized spores, while maintaining a constant supply with fresh medium. When germinated hyphae had reached the measurement chamber, usually about 20–24 h after cultivation start, a varying set of volume flow rates in the range of 0–50 μL min^−1^ was applied. The bending experiments were conducted with the chip holder fixed on the object table of an inverted microscope (*TS200e, Nikon Corporation, Minato, Japan*) and filmed with a high resolution camera (*MikroLive MultiFormat, Schifferstadt, Germany*) equipped with a multi-format CMOS sensor (5472 × 3648 pixels, pixel size = 2.4 µm, *IMX 183, Sony, Tokio, Japan*). In the case of the floor configuration a 60 × objective (*Plan Fluor ELWD, Nikon, Tokyo, Japan*) with extra large working distance, a cover glass correction collar and a numerical aperture NA = 0.7 was used for imaging. For a greater depth of field a 20 × objective (*CFI Achromat LWD, Nikon, Tokyo, Japan*) with NA = 0.4 was used for center configuration measurements. This set up is also depicted in Figure 4B. After conducting the bending tests, the bending stiffness was calculated from the deflection. This was done by extracting the pixel coordinates of the hypha’s central fiber at the wall and at the tip with the image analysis software *FIJI* (Schindelin et al., 2012). To limit the error introduced by the hyphae not growing in the center plane ( z=h/2 ) only hyphae located in close proximity to the center plane were taken into account. This was ensured by only analyzing hyphae that could be focused with sufficient quality in a plane not further away than approximately ± 10 µm from the center plane. The slightly lower flow velocity at this position translates to a maximal deviation from the estimated force in the center plane of less than 5%. Details about the underlying assumptions are given in the provided Supplementary Material file, together with examples of valid and invalid hyphae following the criteria.

#### 2.8.1 From flow to force

**FIGURE 5:**
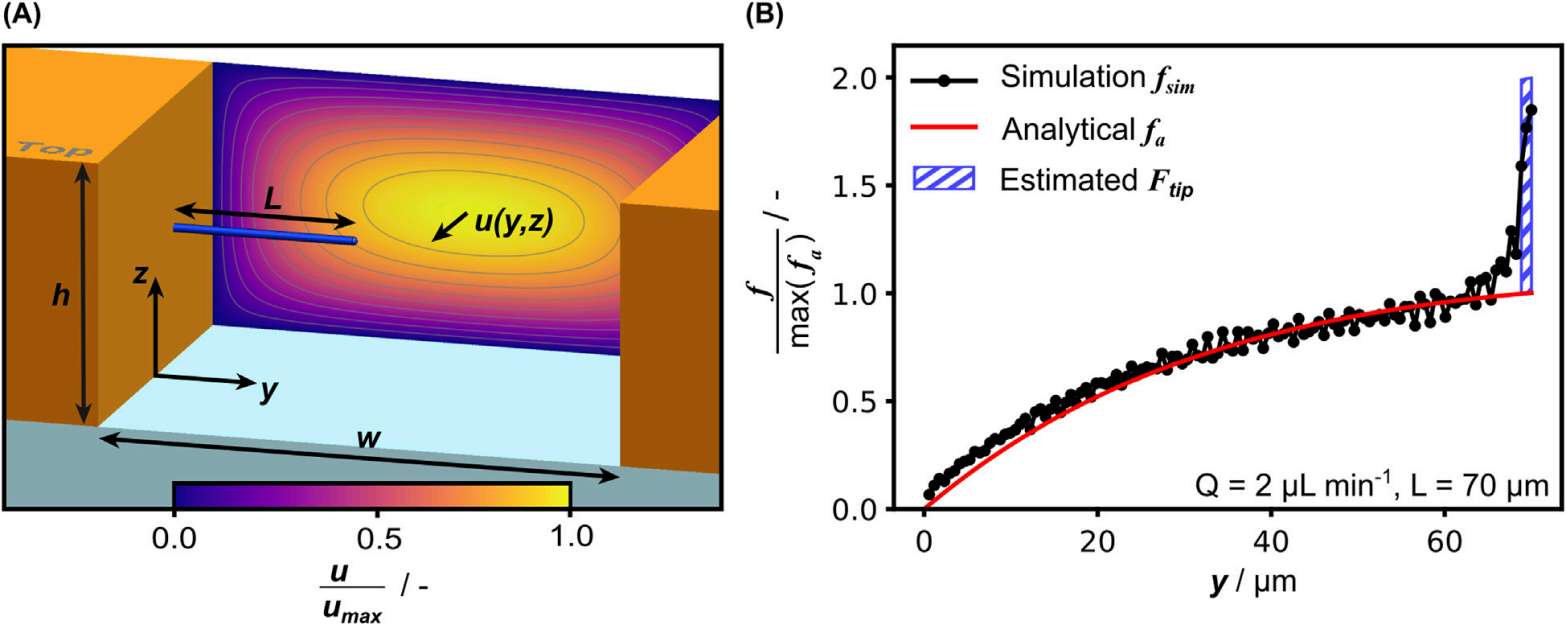
**(A)** Schematic isometric view of the open channel with a hypha in the center configuration and the Poiseuille velocity field in the background. **(B)** Comparison of the force per unit length as calculated with Equation 2 and extracted from CFD simulation results, normalized with the maximum of the analytically calculated value. The integral representation of the estimated $F_{tip}$ calculated with Equation 3 is added as the hatched area.

For calculation of the fluid-induced forces, we considered the hypha inside the measurement chamber of width *w* and height *h* as sketched in Figure 5A. Treating the hypha as a rigid circular cylinder, the flow-induced forces can be derived from the Stokes equations (Lamb, 1911). In the case of microfluidic bending tests in floor configuration, the analytical solution for a cylinder lying on a rigid surface oriented perpendicular to flow direction has been used to calculate the flow-induced force (Schubert, 1967; Amir et al., 2014; Caspi, 2014; Couttenier et al., 2022). The force is proportional to the unperturbed velocity gradient in *z*-direction at the wall $\frac{\delta u}{\delta z}{|}_{z\to 0}$ . However, for the exact progression of the force distribution along a hypha in a microfluidic channel flow, it is necessary to consider its location in the two-dimensional velocity field $u\left(y,z\right)$ and the influence of the channel walls. For this purpose, we calculated magnitude and progression of the fluid induced force by applying the solution of the Poiseuille flow velocity field in a rectangular channel (see SI, Supplementary Equation S5). Substituting this velocity profile accordingly into the analytical 2D solution for the force per unit length, for the hypha in the floor configuration it is given by

$$f_{floor}\left(y\right)=\frac{\;16\;Q\;R_{f}\;h\;r}{\pi }\;\sum_{n=1,3,5,\ldots }^{\infty }\frac{1}{n^{2}}\;\left(1-\frac{\cosh\left(\frac{n\;\pi }{h}\left(y-\frac{w}{2}\right)\right)}{\cosh\left(\frac{n\;\pi \;w}{2\;h}\right)}\right)$$ (1)

with the volume flow rate Q *,* the hyphal radius *r* and the fluidic resistance per unit length $R_{f}$ (formula see Supporting Informations (SI)). The latter also includes the fluid’s dynamic viscosity. Similarly, for the center configuration, the theoretical flow profile has been substituted into the analytical 2D solution for an infinite cylinder submerged in a confined flow in the middle of two parallel plates to yield the force per unit length as

$$f_{center}\left(y\right)=\frac{\;16\;Q{\;R}_{f}\;h^{2}\;\varepsilon }{\pi^{2}}\sum_{n=1,3,5,\ldots }^{\infty }\frac{1}{n^{3}}\;\left(1-\frac{\cosh\left(\frac{n\;\pi }{h}\left(y-\frac{w}{2}\right)\right)}{\cosh\left(\frac{n\;\pi \;w}{2\;h}\right)}\right)\sin\left(\frac{n\;\pi }{2}\right)$$ (2)

where $\varepsilon ={\left(\ln\left(\frac{h}{2\;r}\right)-0.92\right)}^{-1}$ (Harper and Chang, 1967). A detailed derivation of Equations 1, 2 is given in the Supplementary Material (SI). In both cases the infinite series is approximated by considering terms up to *n* = 11 when applied, as terms for higher *n* do practically not contribute anymore (less than 0.5%). While the forces calculated with this analytical model correspond well with simulation data in the cylindrical section of the hypha, the comparison of simulated and calculated f also reveals a non-negligible influence of the hemispherical tip (see Figure 5B). This pronounced end effect for a hemispherical tip has been shown numerically before (Jensen et al., 2016) and leads to a significant increase of the simulated force per unit length at the tip. As the span of this additional load component is small compared to the hyphal length ( L≫r ), this was taken into account as an additional point load $F_{tip}$ at the tip. Based on the simulation results its magnitude was estimated as

$$F_{tip}=\int_{\left(L-r\right)}^{L}f\left(y\right)dy$$ (3)

to effectively double the force per unit length along the hyphal tip. The integral representation is also shown in Figure 5B.

#### 2.8.2 Calculation of the bending stiffness

**FIGURE 6:**
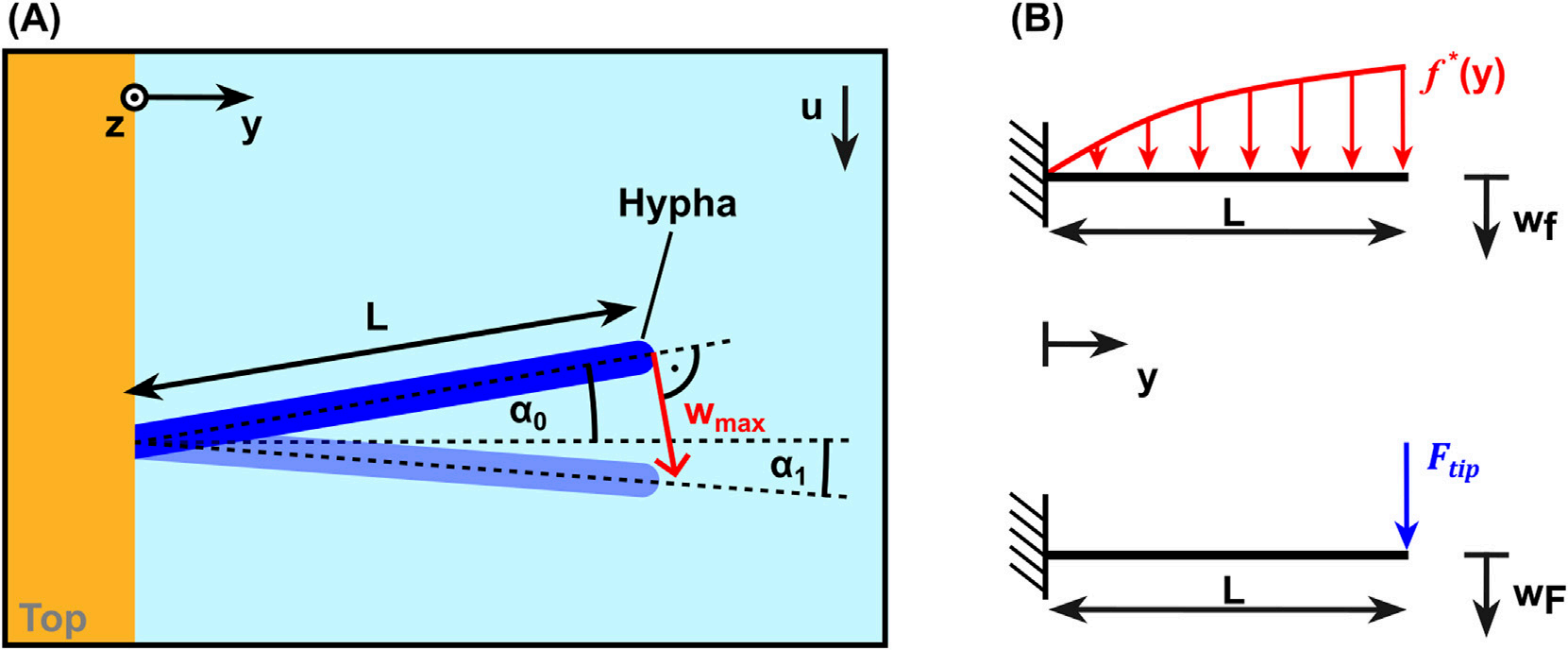
**(A)** Schematic top view of a hypha without flow (dark blue) and in bent state (light blue) defining parameters used for data analysis. **(B)** Schematic free body diagrams for the loads considered: the analytical force per unit length on a cylinder (above) and the estimated tip force (below).

The Euler-Bernoulli beam theory for a beam with constant cross section was applied to calculate the hyphal bending stiffness from the measured tip displacement and the calculated hydrodynamic loads. For this purpose the initial angle $\alpha_{0}$ and the deflected angle $\alpha_{1}$ of every individual hypha were considered, see Figure 6A. The real initial and deflected shape of each hypha is approximated by a straight connection line reaching from its base to its tip. In the deflected state of equilibrium, the hypha is bent by the hydrodynamic load $f^{*}\left(y\right)$ , which compared to $f\left(y\right)$ is diminished by the factor $\cos\left(\alpha_{1}\right)$ due to the force not acting perpendicular to the hypha. As the vertical displacement (perpendicular to the hyphal main axis) is addressed in the Euler-Bernoulli beam theory, the initial tilt angle α_0_ has to be considered to yield the vertical tip deflection from measurements. This is achieved by multiplying the measured displacement in *x*-direction by $\cos{\left(\alpha_{0}\right)}^{-1}$ . The hypha is moreover assumed to be constant in cross-section. With these considerations, the differential equation for the hypha bending problem according to the Euler-Bernoulli beam theory

$$\frac{d^{4}}{{dy}^{4}}\;w_{f}\left(y\right)=\frac{f^{*}\left(y\right)}{k_{b}}=\frac{\cos\left(\alpha_{1}\right)f\left(y\right)}{k_{b}}$$ (4)

with the bending stiffness $k_{b}$ was analytically solved with the python package *SymPy* (Meurer et al., 2017) to yield the solution for the vertical deflection $w_{f}\left(\;y\right)$ due to the distributed load. Depending on the used system and the resulting measurement configuration, either Equation 1 or Equation 2 were used for $f\left(y\right)$ . The boundary conditions were chosen as a fixed support at y=0 and a free end at y=L corresponding to a cantilever beam. To account for the additional tip force $F_{tip}$ , the tip deflection due to the distributed load calculated with Equation 4 was superimposed with the deflection due to a corresponding point load at its tip. Schematic free body diagrams of both load cases are shown in Figure 6B. The resulting relation for the total tip deflection

$$w_{\max}=w_{f,\max}+w_{F,\max}=w_{f,\max}+\frac{F_{tip}L^{3}}{3k_{b}\;}$$ (5)

was used to deduce the bending stiffness from observed tip deflections, where $F_{tip}$ was calculated according to Equation 3 with $f=f^{*}$ . Not considering the tip force leads to an under estimation of the bending stiffness of more than 12% for L = 40 μm, sharply increasing with decreasing hyphal length. To ensure a vanishing relative error introduced by using the linearized small deflection Euler-Bernoulli beam theory, we only considered data points with a relative tip deflection of $w_{\max}/L\leq 0.25$ .

## 3 Results

### 3.1 Spore immobilization and on chip hyphal growth

**FIGURE 7:**
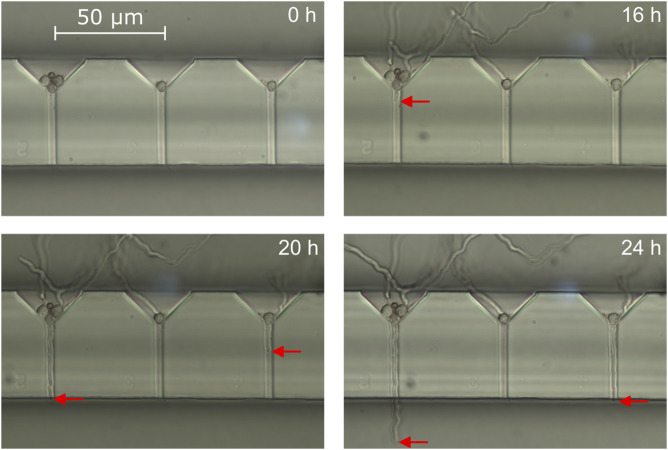
Light microscopy images of three growth channels in a system in floor configuration showing immobilized spores after 0, 16, 20 and 24 h of incubation. The red arrow marks the position of the hyphal tips growing through the channels towards the measurement area.

Before conducting bending experiments, spores were introduced into the system and immobilized at the entrances of the growth channels. With a loading flow rate of 108 μL min^−1^ and a spore concentration of 10^6^ spores mL^−1^, an immobilization rate of 85% was achieved (number of cell traps filled with at least one spore vs. total trap number). The germination and growth process of the hyphae was monitored via white light microscopy in time steps of 4 h while minimizing the time the system was taken out of the incubator. Figure 7 shows representative results of this loading step and the subsequent incubation. Although most of the loaded traps had at least one spore that germinate during the experiment, in this representative case only a small fraction of 6 in total grew into the growth channels and reached the measurement area within the first 24 h. Most spores germinated and grew towards the loading chamber instead of the growth channels, which might be attributed to the small incubation flow of fresh minimal medium both chambers where constantly infused with. As stated in section 2.8, we chose this to prevent dislodging of the immobilized spores. After 42 h, 40 hyphae had reached the measurement area, while 48 hyphae had reached the measurement area by the end of the cultivation (t = 52 h). This delayed strong increase in number of hyphae entering the measurement chamber can be attributed to the germination behavior of *A. niger* conidia. When exposed to favourable conditions after a certain time delay, the conidia first undergo isotropic swelling before germinating later (Meyer et al., 2015). In fact, the swelling can be observed in Figure 7 as a subtle increase of the spore diameters during the first 16 h of incubation. The germination time from cultivation start however differs from spore to spore, leading to a pronounced s-shape of the plotted cumulative number of germinated hyphae over time (Ijadpanahsaravi et al., 2022). While all spores initially came into contact with liquid growth medium at the same time, some thus germinated much earlier than most others. This lead to these few early hyphae growing long distances in the measurement area before others even reached it, later interfering with bending experiments of younger hyphae. The average growth rate inside the growth channels derived from images such as those depicted in Figure 7 is 5.34 ± 2.63 μm h^−1^, meaning by average it took a hypha about 7.5 h to reach the measurement chamber after germination or entering the growth channel.

### 3.2 Determination of flow-induced force

**FIGURE 8:**
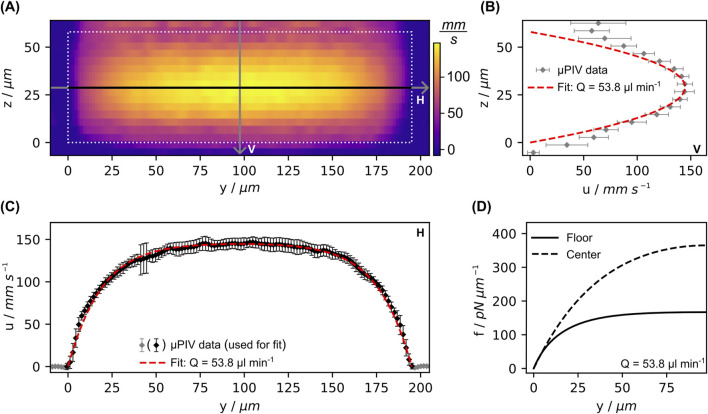
Results from µPIV measurements in the measurement chamber. **(A)** Full measured velocity field (x-component) over the cross-section of the measurement chamber half way between in- and outlet. **(B, C)** provide thereof extracted profiles vertically (V) and horizontally (H) through the center, as indicated by the labeled lines in **(A)**. **(D)** Progression of the distributed force calculated from the fit for both configurations, for equations see SI. Error bars indicate the standard deviation of the average along *x*-direction.

In order to verify the theoretical flow profile and calculate the corresponding flow-induced forces, the entire flow field over the cross-section was investigated via µPIV. In Figure 8A the results of µPIV measurements of the flow velocity profile in the measurement chamber are depicted. Together with the knowledge about the actual chamber cross-section dimensions acquired from microscopy measurements (data not shown), this data was used to fit the analytical solution for the Poiseuille velocity field in a channel with rectangular cross-section. Although the measured horizontal profile over the chamber width agrees well with the theoretical profile (see Figure 8C), the vertical profile over the channel height in Figure 8B shows significant deviations near the horizontal walls. The velocity does not reach zero but remains at approximately 30% of the maximum velocity at the center. The reason for this lies in the field of depth, which is not restricted to a defined focal plane. Even particles that are just out of focus and therefore slightly blurred are still captured. This means that flowing particles are still detected even though the focal plane is already outside the chamber (Rossi et al., 2012). Therefore, we used the volume flow rate derived from the fit to the horizontal profile in Figure 8C to calculate the fluid-induced force per unit length. Its progression is shown in Figure 8D for both configurations, where the maximal achieved force per unit length at y=w/2 is approximately 160 pN μm^−1^ in floor configuration and 380 pN μm^−1^ in center configuration. While f is approximately proportional to u for the center configuration, in floor configuration it reaches a plateau after about w/4 due to the proportionality to the wall velocity gradient $\frac{\delta u}{\delta z}{|}_{z\to 0}$ . The portrayed load progression for the center configuration stems from the chamber cross section with double the height as compared to the floor configuration, which results in half of the average velocity at the same flow rate. We chose this because these are the actual geometrical parameters of the systems used. Comparing the measurement results close to the entrance and at the exit of the measurement chamber showed no significant variation of velocity along the flow direction (data not shown), thus any possible influence of the growth channels on the flow field along the length of the measurement chamber were assumed to be negligible.

### 3.3 Measurement of the bending stiffness

**FIGURE 9:**
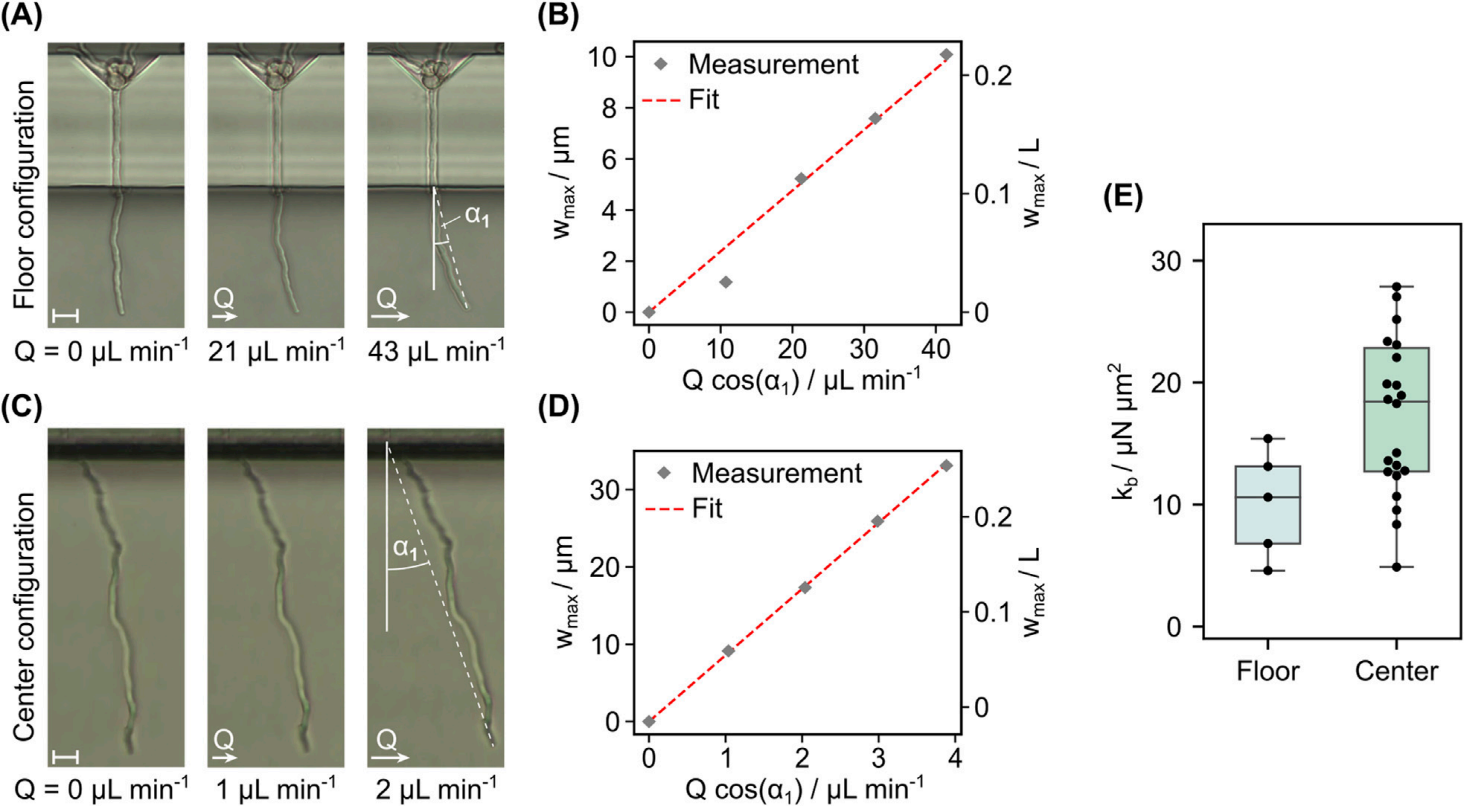
Results from bending experiments for floor and center configuration. **(A)** Frames recorded during a bending experiment with increasing flow rates in floor configuration and **(B)** values of tip deflection extracted from these frames versus the applied volume flow rate scaled by the equilibrium tilt angle. The fit used to extract $k_{b}$ is indicated by the red dash-line. **(C, D)** show the respective frames and plotted data for a hypha in center configuration. In **(C)**, the cell traps and growth channels are located above the image section shown, with the growth channel exits visible at the very top. **(E)** Box plots of the measured bending stiffness of all analyzed hyphae in floor (n_floor_ = 4 from two cultivations) and center configuration (n_center_ = 22 from one cultivation, one outlier with $k_{b}$ = 45 μN μm^2^ not shown). Scale bars = 10 µm.

When the first hyphae had reached the measurement chamber such that a minimal hyphal length of 30 µm was exposed, bending flows were applied to measure the bending stiffness. In Figure 9A, the exemplary results of a bending test in floor configuration at different flow rates are shown. The depicted hypha was exposed along a length of 46 µm. For every volume flow rate the tip deflection derived from the microscopy images is plotted in Figure 9B. With the assumptions discussed earlier, the term $Q\;\cos\left(\alpha_{1}\right)$ on the horizontal axis is proportional to the hydrodynamic forces and the hyphal tip deflection. The proportionality factor includes the hyphal length and the bending stiffness. In this example, the displayed fit of Equation 5 to yield the bending stiffness is consistent with the data, except for the deflection value at the lowest volume flow rate. This could be accounted to an initial partial adhesion to the glass surface, while the increasing volume flow rates applied afterwards were sufficient to loosen the hypha permanently. In fact, due to their often strong adhesion to the glass surface in floor configuration, many hyphae did not show any deflection, even at high volume flow rates. Only hyphae without such obvious attachment to the glass surface were taken into account for analysis, leading to low data yields despite the high amount of hyphae in the measurement chamber. Similarly, hyphae that grew out of the measurement plane, which manifested in defocusing of these parts of the cells, were also not included in the analysis. With these criteria, in floor configuration the bending stiffness of only five hyphae could be measured during two cultivation experiments, obtaining a mean bending stiffness of 10.9 ± 4.5 μN μm^2^. The symmetrical system design in center configuration, that was developed to avoid the aforementioned problems, was used in an analogous experiment. Exemplary results for a hypha with a length of 134 µm in center configuration are shown in Figures 9C, D, while in Figure 9E the results of bending tests for both configurations are compared. It should be noted that the flow rates necessary to induce the same amount of deflection for both displayed hyphae differed by a factor of approximately 40 from center to floor configuration. This is mainly due to the higher induced force per unit length for the same flow rate in the central configuration (see Figure 8D) and the strong dependence of the tip deflection on the exposed hyphal length. Nevertheless, as the comparison of the results for both configurations demonstrates, the extracted bending stiffness still is in the same order of magnitude. Also, the meandering growth path observed in Figure 9C is representative for other hyphae, especially in center configuration. A possible explanation for this is the small but non-vanishing flow induced force due to the incubation flow imposed by the syringe pumps, which most likely also expresses a slight rippling profile. Additionally, the growth behavior is generally influenced by the genetics of the strain and the growth conditions (Aleklett et al., 2021). For the center configuration, we obtained a slightly higher mean bending stiffness of 18.3 ± 8.7 μN μm^2^ as compared to the floor configuration. For the latter, the displayed results were obtained over the course of two cultivations, while for the center configuration all displayed data was obtained from a single cultivation experiment with only one microfluidic chip. This vastly improved yield for the center configuration reflects the absence of chamber floor adhesion due to the symmetrical measurement setup. None of the analyzed hyphae showed significant hysteresis when returning to their initial shape after the measurement flow vanished. The reason for the approximately 66% increase in measured bending stiffness in between the configurations could be a systematic underestimation of the flow induced forces in floor configuration. To illustrate, due to the hyphae deviating from the ideal case of full contact with the underlying surface over its length, flow between the hypha and surface might occur, significantly increasing drag. Conversely, in the center configuration, an overestimation of the forces due to the hyphae growing away from the center plane of maximum velocity ( z=h/2 ) would in turn lead to an overestimation of the bending stiffness.

## 4 Discussion

The definition of the second moment of inertia for a circular ring cross section can be used to calculate the corresponding longitudinal Young’s Modulus of the hyphal cell wall E from the measured bending stiffness. Transmission electron microscopy (TEM) measurements of the cross section of *A. niger* SKAN1015 hyphae reveal an average wall thickness of 287 nm (see Supplementary Figure S1). Together with a measured average hyphal radius of 1.4 µm this yields a Young’s Modulus of 10.1 ± 4.5 MPa for the hyphal cell wall of the strain SKAN1015 in center configuration. Details about the calculations can be found in the SI. This value is higher than the value reported for *C. albicans* hyphae also measured through microfluidic bending tests, but in floor configuration. For reference, using the few data points we could record in floor configuration yields a Young’s Modulus of 5.5 MPa, which is very close to the reported value for *C. albicans* (Couttenier et al., 2022). Carefully comparing the effective indentation modulus for *A. niger* hyphal cell wall obtained by AFM measurements of a different strain show that they are generally higher, but in the same order of magnitude at comparable conditions (Günther, 2015; Zheng et al., 2017). However, it should be noted that in both cases the measurements were conducted with specimen grown at a slightly lower temperature of 30°C. Additionally, the results of the ordinary least squares regression analysis of the dataset indicate a significant linear dependency of the bending stiffness on the exposed hyphal length ( $P<\;10^{-4}$ , see Supplementary Figure S4 in SI). A possible explanation for this could be the inevitably older average age of the cell wall of longer hyphae. Thus, for longer hyphae the average age of the cell wall is older than for shorter hyphae. Zheng et al. (2017) have shown for *A. niger* hyphae that their cell wall’s AFM indentation modulus increases significantly with cultivation time. An increase in cell wall elasticity modulus with cell wall age would then lead to a proportional increase in bending stiffness of these hyphal segments. Our model assumes constant bending stiffness, and thus cell wall elasticity and cross section, along the hyphae, so the measured average bending stiffness in this case would increase, as seen in the hyphae with longer exposed lengths.

## 5 Conclusion

To the authors best knowledge, the presented work is the first reported measurement of the bending stiffness of single *A. niger* hyphae and among few works that tackle their micro-mechanical properties. The here derived elasticity of the cell wall still complies well with those reported by these scarce works (Günther, 2015; Zheng et al., 2017). The presented symmetrical system in center configuration advances the microfluidic bending technique in addressing key shortcomings of the previously published designs (Nezhad et al., 2013; Amir et al., 2014; Caspi, 2014; Couttenier et al., 2022). Due to the positioning of the hypha in the vertical center of the measurement flow, the problem of adhesion or friction to the glass surface is eliminated. This leads to virtually all hyphae growing into the measurement flow being deflectable and thus drastically increases specimen number per cultivation. Despite this significant advantage of the center configuration, a compromise has to be made regarding the in plane growth, as the hyphae lack the growth guidance of the glass surface. However, even though in both configurations hyphae regularly grew out of the measurement plane in *z*-direction, in the case of the center configuration this is less critical to the measured bending stiffness. This is because the actual deviation from the calculated load in the measurement plane is expected to be small for moderate out of plane growth, owing to the low velocity gradient in *z*-direction close to the measurement plane. Thanks to the fabrication of the negative mould using two-photon polymerization, a real-3D conical funnel shape could be realized, yielding a high shape conformity between spherical spore and cell trap. This effectively eliminated the need for any adhesion promoting surface treatment, as it was used by Couttenier et al. (2022) in their systems or the introduction of design alterations, like kinks, in the path of the growth channels to secure hyphal fixation during measurements (Nezhad et al., 2013).

Altogether, the microfluidic bending test in center configuration is a promising tool for parallelized studies of the mechanical properties of filamentous cells, as evidenced by the significant improvement in the measurement yield per experiment compared to the conventional floor configuration design. This enables statistically significant results on the influence of environmental factors on the bending stiffness of the cells within singular on chip cultivation measurements. As opposed to AFM force spectroscopy, the presented system demonstrates the capability to measure cell wall properties without the need for any further handling of the live cells, enabling the non-invasive monitoring of mechanical markers during cultivation. Future work will aim at the application of the methodology to different pharmaceutically relevant species such as actinomycetes for a better understanding of the cultivation process. Another focus will be the integration of sensors into the microfluidic system, such as a passive 2 PP-fabricated flow sensor to aid data analysis as well as the monitoring of the cultivation conditions inside the microsystem. Finally, to be able to study the individual growth morphology in more detail and to incorporate it into the data analysis, we aim at integrating live monitoring of the 3D-shape during tests by combining our implemented protocol with confocal microscopy. In summary, our presented work provides a basis for resolved and detailed on-chip study of filamentous cultures and their mechanics.

## Data Availability

The raw data supporting the conclusions of this article will be made available by the authors, without undue reservation.
