# Supplementary material for: Highly parallel bending tests for fungal hyphae enabled by two-photon polymerization of microfluidic mold
Source: Front Bioeng Biotechnol. 2024 Nov 1;12:1449167. doi: 10.3389/fbioe.2024.1449167 (PMC11563782; doi:10.3389/fbioe.2024.1449167)
Supplement: Supplementary file 1 [file DataSheet1.docx]

**Supplementary Informations: “Highly parallel bending tests for fungal hyphae enabled by two-photon polymerization of microfluidic mold”**

# Flow-induced force calculation

We calculated the flow-induced forces by treating the hyphae as circular cylinders. The 2D-case of the drag force per unit length acting on a circular cylinder located in between two parallel plates at low Reynolds numbers has been studied analytically, numerically and experimentally (Ben Richou et al., 2005). Harper and Chang solved the Stokes equation for the case of a Poiseuille flow profile in between the parallel plates with the cylinder located in the middle of the plates and derived the formula

| $f_{\parallel}=\frac{4\pi}{\ln\left( \frac{h}{2r} \right)-0.92}\mu U_{center}$ | ( S 1 ) |
| --- | --- |

where $\mu$ is the fluid’s dynamic viscosity, $U_{center}$ is the flow velocity in the center in between the parallel plates, $h$ is the distance in between the plates and $r$ is the cylinder radius (Harper and Chang, 1967). The drag force in this case of low flow velocities is proportional to the flow velocity and the fluid’s viscosity, independent from the Reynolds number but a function of the aspect ratio of cylinder and channel height. We used Eq. ( S 1 ) to calculate the induced drag force in the center configuration, with the hyphae located in the vertical channel center. In contrast, for the same cylinder lying on a rigid surface, the drag force per unit length in a constant shear flow is given by

| $f_{s}=4\pi r\mu\frac{\delta u}{\delta z}\left. \right\vert_{z\to0}$ | ( S 2 ) |
| --- | --- |

where the term $\frac{\delta u}{\delta z}\left. \right|_{z\to0}$ is the unperturbed velocity gradient at and normal to the surface (Schubert, 1967). Eq. ( S 2 ) resembles the load case in the floor configuration and has been applied to microfluidic bending of filamentous cells lying on the channel floor to calculate the fluid induced forces (Amir et al., 2014; Caspi, 2014; Couttenier et al., 2022).

For the exact progression of the force distribution along a hypha in a microfluidic channel flow we considered its location in the flow field and the influence of the vertical channel walls. As the Reynolds number of the channel flow $Re_{C}=\frac{\bar{u}\rho d_{h}}{\mu}$ is in the order of unity or lower over all applied flow rates, the flow in the measurement chamber is expected to be laminar. Here the hydraulic diameter of the channel $d_{h}=\frac{2hw}{h+w}$ is used, where $w$ and $h$ are the channel width and height, respectively. When the driving pressure gradient $\frac{dp}{dx}$ as well as the channel cross-section along the *x*-direction are constant, the velocity field developed at these conditions is uniform and only a function of the *y* and *z* positions. It is provided by the Poiseuille flow solution, which, for constant rectangular cross-sections with moderate aspect ratio, such as in the measurement channel, is given by the infinite series

| $u\left( y,z \right)= \frac{4\frac{dp}{dx}h^{2}}{\mu\pi^{3}} \sum_{n=1,3,5,\ldots}^{\infty} \frac{1}{n^{3}}\left( 1-\frac{\cosh\left( \frac{n\pi}{h}\left( y-\frac{w}{2} \right) \right)}{\cosh\left( \frac{n\pi w}{2h} \right)} \right)sin\left( \frac{n\pi z}{h} \right)$ | ( S 3 ) |
| --- | --- |

(Mortensen et al., 2005). The Hagen-Poiseuille law $\frac{dp}{dx}=QR_{f}$ can be applied to substitute the pressure gradient with the fluidic resistance per unit length

| $R_{f}= \frac{12\mu}{h^{3}w} \left( 1-\sum_{n=1,3,5\ldots}^{\infty} \frac{1}{n^{5}}\frac{192h}{{w\pi}^{5}}\tanh\left( \frac{n\pi w}{2h} \right) \right)$ | ( S 4 ) |
| --- | --- |

to yield the flow velocity field

| $u\left( y,z \right)= \frac{4QR_{f}h^{2}}{\mu\pi^{3}} \sum_{n=1,3,5,\ldots}^{\infty} \frac{1}{n^{3}}\left( 1-\frac{\cosh\left( \frac{n\pi}{h}\left( y-\frac{w}{2} \right) \right)}{\cosh\left( \frac{n\pi w}{2h} \right)} \right)sin\left( \frac{n\pi z}{h} \right)$ | ( S 5 ) |
| --- | --- |

as a function of the volume flow rate. Inserting the center velocity along the hypha

| $U_{center}=u\left( y,z=h/2 \right)$ | ( S 6 ) |
| --- | --- |

into Eq. ( S 1 ) yields Eqs. ( 1 ) from the methods section. Similarily, using Eq. ( S 5 ) in Eq. ( S 2 ) yields Eq. ( 2 ) from the methods section. Eq. ( S 5 ) was also used as the fit function to the µPIV measurements.

# Analytical solution for the bending problem

To derive the bending stiffness from observed deflection, the individual hyphae were treated as Euler-Bernoulli beams. According to the Euler-Bernoulli beam theory, the vertical deflection $w(y)$ of a long, slender beam with constant cross section exposed to a distributed load $f\left( y \right)$ along its length $L$ is given by the inhomogenous ordinary differential equation of 4^th^ order

| $\frac{d^{4}}{{dy}^{4}}w_{f}(y)=\frac{f\left( y \right)}{k_{b}}$ | ( S 7 ) |
| --- | --- |

where

| $k_{b}=EI_{z}$ | ( S 8 ) |
| --- | --- |

is the bending stiffness constituted of the Young’s modulus $E$ of the material, here being the cell wall, and the second moment of area of its cross section with respect to the bending axis $I_{z}$ (Gere and Goodno, 2012). By applying the boundary conditions for a cantilever beam fixed at one end

| $w_{f}\left( y=0 \right)=0$ | ( S 9 ) |  |
| --- | --- | --- |
| $\frac{d}{dy}w_{f}\left( y=0 \right)=0$ | ( S 10 ) |  |
| $\frac{d^{2}}{dy^{2}}w_{f}\left( y=L \right)=0$ | ( S 11 ) |  |
| $\frac{d^{3}}{dy^{3}}w_{f}\left( y=L \right)=0$ | ( S 12 ) |  |

We solved the beam equation with the aid of the *SymPy* python package to yield the solution for the vertical tip deflection due to the distributed load $w_{f,max}=w_{f}\left( y=L \right)$. Due to the inherent linearity of the Euler-Bernoulli beam theory, assuming small deflections relative to the beam’s length, the additional load corresponding to the tip force $F_{tip}$ can be treated separately according to the principle of superposition. Therefore, the well known solution for the tip deflection of a cantilever induced by a concentrated force at its tip (Gere and Goodno, 2012)

| $w_{F}\left( y=L \right)=w_{F,max}=\frac{F_{tip}L^{3}}{{3k}_{b}}$ | ( S 13 ) |
| --- | --- |

can be added to the deflection induced by the distributed load to yield a relation for the total observed deflection. The choice of boundary conditions corresponds to the hyphae being clamped at the exit of the growth channel, which is justified by the tight fit of the growth channel’s and the hyphal cross-sections. We observed no lateral movement of the hyphae at the exit of the growth channel during bending tests.

# Transmission electron microscopy of hyphal cross-sections

The hyphal cell wall thickness of the *A. niger* strain SKAn1015, which was also used for the bending tests, was studied by transmission electron microscopy (TEM). For this prupose, the fixative (480 μL of 25% glutaraldehyde (GA) + 1200 μL of 25% paraformaldehyde (PFA)) was directly added to 4320 μL of hyphae cultures, resulting in a final concentration of 5% PFA and 2% GA. The hyphae were incubated overnight at room temperature. The fixed samples were washed twice with 0.1 M EM-HEPES buffer solution and then incubated in osmium tetroxide (1% in HEPES buffer) for one hour at room temperature. This was followed by another washing step with HEPES buffer solution. The dehydration of the hyphae was performed with a gradual series of ethanol (10, 30, 50, 70, and 90%) for 30 minutes each, with the 70% ethanol step at 4°C and the addition of 2% uranyl acetate overnight. Finally, two dehydration steps in 100% ethanol were performed at room temperature for 30 minutes each. The cells were then infiltrated with LR White acrylic resin (LRW) in a gradual series (mixing ratio LRW/Ethanol: 1:1, 2:1, 2x 100% LRW). Each step was incubated overnight at room temperature. After the addition of the catalyst, the polymerization of the acrylic resin occurred at 50°C for two days. Ultrathin sections of 50-70 nm thickness were made from the samples using an ultramicrotome (Ultracut, Reichert). This was followed by contrast staining with 4% aqueous uranyl acetate for 3 minutes and lead citrate for 15 seconds. Images were taken with a Libra 120 Plus (*Zeiss, Oberkochen, Germany*), at an acceleration voltage of 120 kV and calibrated magnification. A representative TEM image of a hyphal cross-section is shown in Figure S 2.

# Calculation of cell wall Young’s Modulus

| $I_{z}=\frac{\pi}{4}\left( r^{4}-\left( r-t_{w} \right)^{4} \right)$ | ( S 14 ) |
| --- | --- |

The definition of the bending stiffness from Eq. ( S 8 ) was used to calculate the cell wall’s longitudinal Young’s modulus $E$ in assuming the hyphae as circular tubes. In that case the second moment of area with respect to the bending axis (z‑axis) is

with the wall thickness $t_{w}$. From TEM images of hyphal cross-sections we measured an average hyphal cell wall diameter of 287 nm (n = 8), while the average hyphal radius directly derived from white light microscope images of the on-chip culture was 1.4 µm. Using this information we calculated the cell walls longitudinal Young’s modulus by using ( S 14 ) in ( S 8 ), solving for $E$.

# Estimation of the maximum deviation of the hyphal focal planes

As hyphae usually did not grew perfectly straight in the center plane, to limit errors arising from false estimation of the forces a specimen had to fulfill two criteria: Firstly, a sufficiently in plane growth such that most of the hypha could be focused in one focal plane. This limited the out of z-plane tilt by the depth of field (*DoF*) of the imaging system. The latter was calculated by

| $DoF=\frac{n_{m} \lambda}{\text{NA}^{2}}+ \frac{n_{m} e}{M \text{NA}}$ | ( S 15 ) |
| --- | --- |

with the refractive index of the medium $n_{m}$ (here $n_{m}$ = 1.33 for water at 21°C), the mean wavelength of the light $\lambda$ (here $\lambda=550 nm$ for white light), the numerical aperture $\text{NA}$ and Magnification $M$ of the objective and the pixel size of the sensor $e$ (Pawley, 2006). For the 20x objective used in center configuration this evaluates to a depth of field of approximately 5.0 µm. Secondly, the maximum deviation of the focal plane during experiments from the center ($z=h/2$) was limited by the fact that the spores and growth channels still had to be reasonably in focus at the same time. Figure S 2 gives two examples of a valid and a non-valid hypha according to these criteria. Together these considerations give a z-deviation of the hyphal growth plane from the center of approximately $\pm$10 µm. The flow velocity at $z=h/2\pm10$µm is still more than 95% of the center velocity. As the flow induced force is proportional to the flow velocity, it can be argued that with less than 5% the error introduced by this deviation is tolerable. Even for a deviation of up to $\pm15$ µm the error remains below 10%.


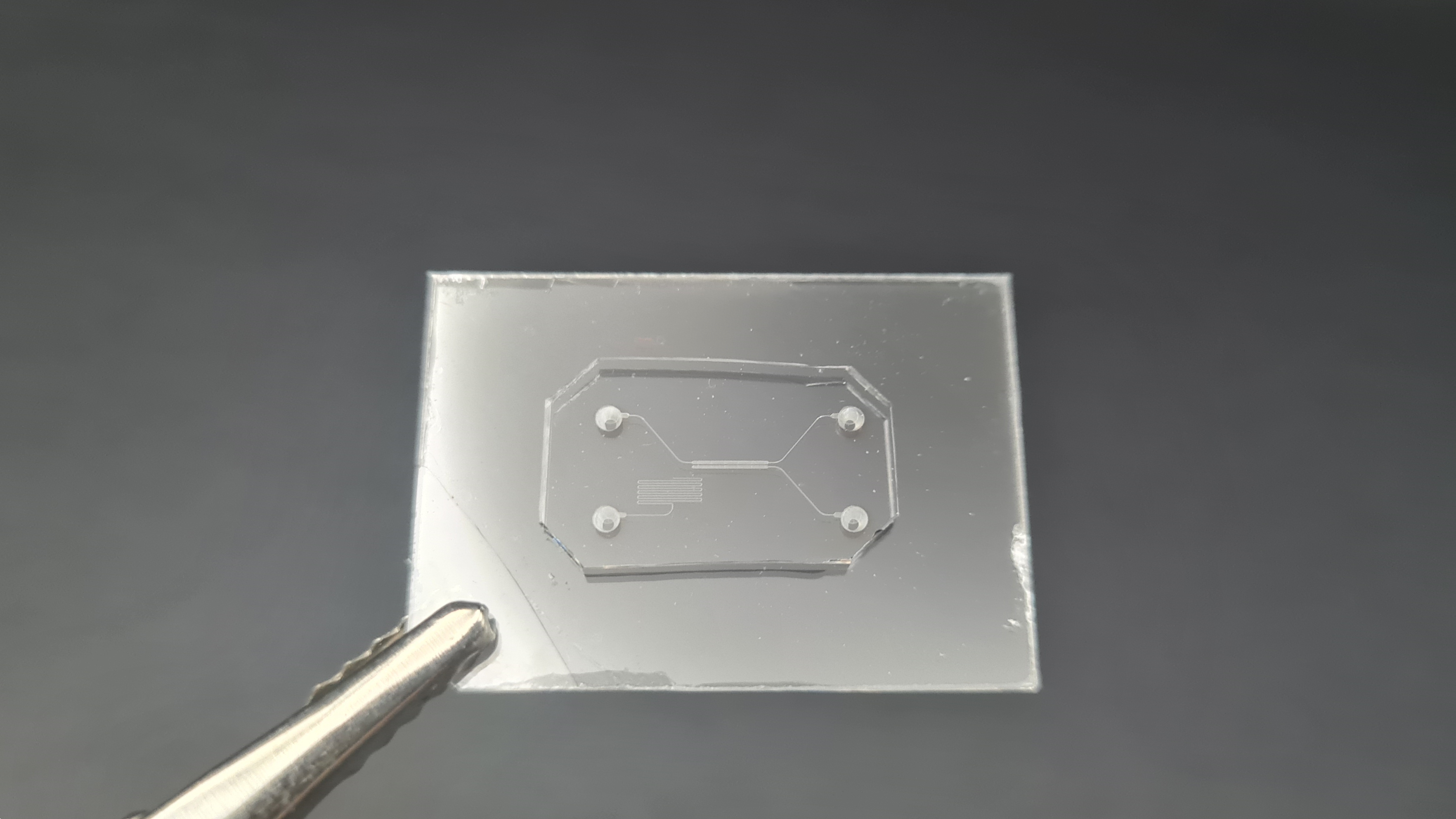


Figure S 1: Photograph of an assembled system in center configuration.


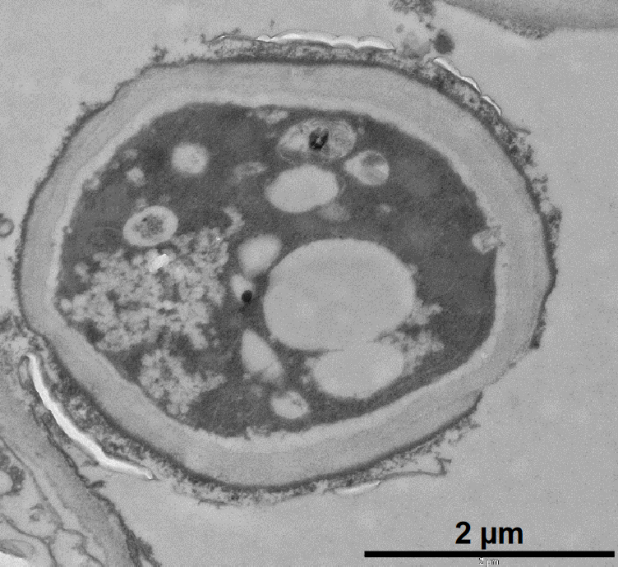


**Figure S 2:** TEM image of the cross‑section of a hypha of *A. niger* strain SKAn1015


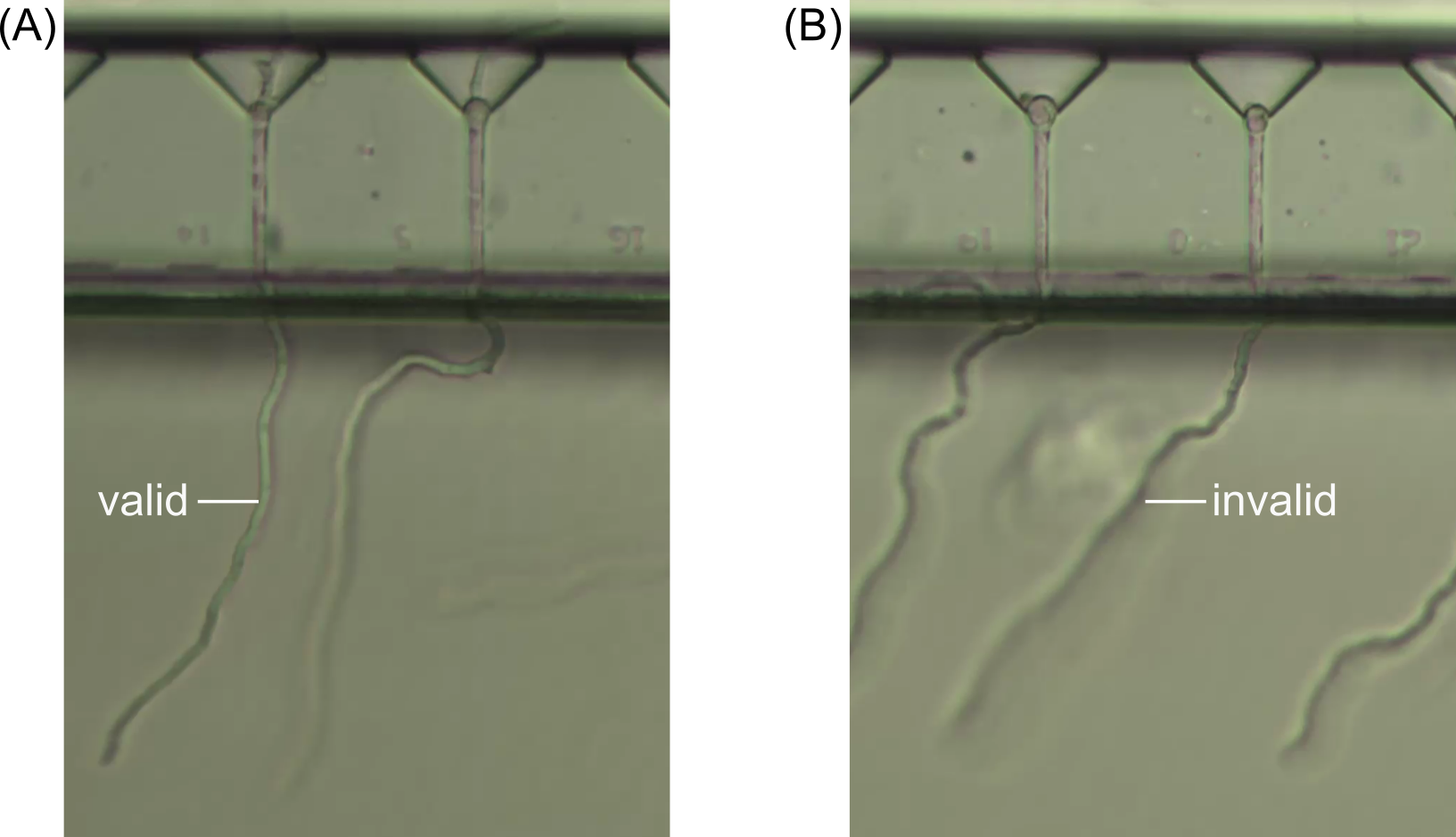


Figure S 3: Examples for specimen in center configuration that fulfilled the z-deviation criteria and that did not. (A) Only the left hypha has regular growth and is focused in a plane where also the spore and the growth channel are. Therefore, it is valid for measurement. (B) The hyphae express strong out of plane growth and therefore where not accounted for.


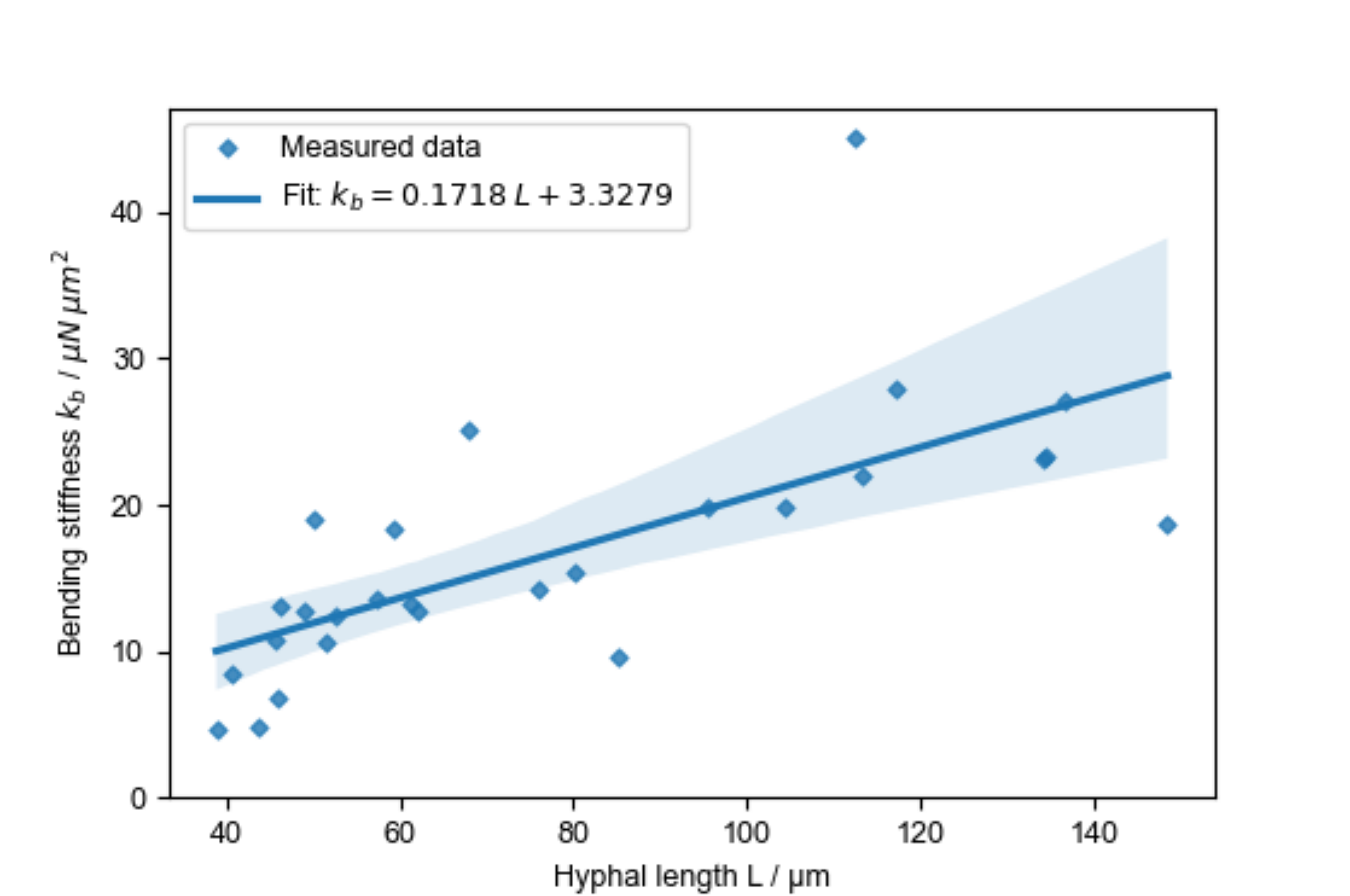


**Figure S 4:** Measured bending stiffness over the hyphal length for all measurements. The Ordinary Least Square regression depicted by the fit implies a significant dependency of the bending stiffness on the hyphal length in our data set (p-value $<{10}^{-4}$).

# References

Amir, A., Babaeipour, F., McIntosh, D. B., Nelson, D. R., and Jun, S. (2014). Bending forces plastically deform growing bacterial cell walls. *Proceedings of the National Academy of Sciences of the United States of America* 111, 5778–5783. doi: 10.1073/pnas.1317497111

Ben Richou, A., Ambari, A., Lebey, M., and Naciri, J. K. (2005). Drag force on a circular cylinder midway between two parallel plates at Re≪1 Part 2: moving uniformly (numerical and experimental). *Chemical Engineering Science* 60, 2535–2543. doi: 10.1016/j.ces.2004.08.050

Caspi, Y. (2014). Deformation of filamentous Escherichia coli cells in a microfluidic device: a new technique to study cell mechanics. *PloS one* 9, e83775. doi: 10.1371/journal.pone.0083775

Couttenier, E., Bachellier-Bassi, S., d’Enfert, C., and Villard, C. (2022). Bending stiffness of Candida albicans hyphae as a proxy of cell wall properties. *Lab Chip* 22, 3898–3909. doi: 10.1039/D2LC00219A

Gere, J. M., and Goodno, B. J. (2012). *Mechanics of Materials*. Cengage Learning.

Harper, E. Y., and Chang, I. ‐Dee (1967). Drag on a Cylinder between Parallel Walls in Stokes’ Flow. *The Physics of Fluids* 10, 83–88. doi: 10.1063/1.1761999

Mortensen, N. A., Okkels, F., and Bruus, H. (2005). Reexamination of Hagen-Poiseuille flow: Shape dependence of the hydraulic resistance in microchannels. *Phys. Rev. E* 71, 057301. doi: 10.1103/PhysRevE.71.057301

Pawley, J. B. ed. (2006). *Handbook Of Biological Confocal Microscopy*. Boston, MA: Springer US. doi: 10.1007/978-0-387-45524-2

Schubert, G. (1967). Viscous flow near a cusped corner. *Journal of Fluid Mechanics* 27, 647–656. doi: 10.1017/S0022112067002526
